# BAC transgenic mice to study the expression of P2X2 and P2Y_1_ receptors

**DOI:** 10.1007/s11302-021-09792-9

**Published:** 2021-05-28

**Authors:** Marcus Grohmann, Michaela Schumacher, Janka Günther, Stefan M. Singheiser, Tanja Nußbaum, Florian Wildner, Zoltan Gerevich, Ronald Jabs, Daniela Hirnet, Christian Lohr, Peter Illes, Günther Schmalzing, Heike Franke, Ralf Hausmann

**Affiliations:** 1 Rudolf Boehm Institute of Pharmacology and Toxicology, University of Leipzig, Leipzig, Germany; 2 Present Address: HTK Hygiene Technologie Kompetenzzentrum GmbH, Bamberg, Germany; 3 Institute of Clinical Pharmacology, RWTH Aachen University, Wendlingweg 2, 52074 Aachen, Germany; 4 Institute of Neurophysiology, Charité-Universitätsmedizin Berlin, Berlin, Germany; 5 Institute of Cellular Neurosciences, Medical Faculty, University of Bonn, Bonn, Germany; 6 Division of Neurophysiology, University of Hamburg, Hamburg, Germany

**Keywords:** P2X2 receptor, P2Y_1_ receptor, Brain, TagRFP, BAC transgenic mice

## Abstract

Extracellular purines are important signaling molecules involved in numerous physiological and pathological processes via the activation of P2 receptors. Information about the spatial and temporal P2 receptor (P2R) expression and its regulation remains crucial for the understanding of the role of P2Rs in health and disease. To identify cells carrying P2X2Rs in situ, we have generated BAC transgenic mice that express the P2X2R subunits as fluorescent fusion protein (P2X2-TagRFP). In addition, we generated a BAC P2Y_1_R TagRFP reporter mouse expressing a TagRFP reporter for the P2RY1 gene expression. We demonstrate expression of the P2X2R in a subset of DRG neurons, the brain stem, the hippocampus, as well as on Purkinje neurons of the cerebellum. However, the weak fluorescence intensity in our P2X2R-TagRFP mouse precluded tracking of living cells. Our P2Y_1_R reporter mice confirmed the widespread expression of the P2RY1 gene in the CNS and indicate for the first time P2RY1 gene expression in mouse Purkinje cells, which so far has only been described in rats and humans. Our P2R transgenic models have advanced the understanding of purinergic transmission, but BAC transgenic models appeared not always to be straightforward and permanent reliable. We noticed a loss of fluorescence intensity, which depended on the number of progeny generations. These problems are discussed and may help to provide more successful animal models, even if in future more versatile and adaptable nuclease-mediated genome-editing techniques will be the methods of choice.

**Supplementary Information:**

The online version contains supplementary material available at 10.1007/s11302-021-09792-9.

## Introduction

The functions of the purine nucleotide ATP as a potent extracellular neurotransmitter via the activation of the P2 receptor (P2R) family is well-established, and virtually every tissue expresses P2Rs, indicating that ATP and related purines act as a common transmitter in almost all tissues and organs [1]. Information about the precise distribution of specific receptor subtypes on specific cells such as neurons or glial cells and their regulation in disease models remains crucial for the understanding of the role of P2R-mediated cellular processing in health and disease (comprehensively reviewed in [1]).

P2XRs are homo- and heterotrimeric cation-selective ligand-gated ion channels, which open upon extracellular binding of ATP and thus act as electrical “cell surface ATP sensors” [2]. P2XRs are involved in a variety of physiological functions throughout the body, including neuroeffector transmission from neuron to muscle, neurotransmission from neuron to neuron, bladder function, and breathing or taste perception (for review see [3–5].

The P2X2R is one of the most widely expressed P2XRs in the body. P2X2R subunits have been found in the central (CNS) and peripheral (PNS) nervous systems [1], with high expression levels in the cerebral cortex, basal ganglia, diencephalon, mesencephalon, cerebellum, medulla oblongata, olfactory bulb, and dorsal horn of the spinal cord [6] and the inner ear [5]. Despite its widespread CNS expression, P2X2 knockout mice do not exhibit a strong CNS phenotype in terms of general excitability and sensory and motor function [4, 7]. Possible explanations are that P2X2Rs are of secondary importance for the healthy brain or that other genes functionally compensate their deletion. The involvement of P2X2R subunit-containing receptors in sensory transmission has been confirmed in acute and chronic pain models performed with P2X2R subunit knockout mice [7]; for review, see [8].

P2YRs are a family of G protein-coupled receptors [9], which are known to be expressed in almost all mammalian tissues (for review see [9]). P2YRs mediate responses such as vasodilation, blood clotting/platelet aggregation, and immune response [9]. Clopidogrel, a blockbuster drug that has been used for thrombosis prophylaxis since 1996, was found in 2001 to exert its antithrombotic effects by irreversibly blocking the P2Y_12_R on platelets through an active metabolite formed in the liver [10]. Other P2YRs are also common targets in drug development [9, 11]. In the CNS, P2Y_1_Rs are found on neurons, e.g., of the hippocampus, basal ganglia, cerebellum, and spinal cord. For instance, P2Y_1_Rs are thought to be involved in proliferation and migration processes of neuronal progenitor cells, in brain development and regeneration, and in sensory neurotransmission and pain perception [12–17].

Taken together, P2X2- and P2Y_1_Rs are crucially involved in neuronal and glial physiology. There is also accumulating evidence indicating a role of these receptors under diverse pathophysiological conditions and disorders of the CNS [18]. In particular, there is converging evidence from electrophysiological, genetic, and pharmacological studies supporting an important role for P2X2-, P2X3-, and P2Y_1_Rs in nociception [8, 19, 20]. Accordingly, P2X/PYRs have attracted widespread interest as novel therapeutic targets (for recent reviews see [5, 9]), which has led to the “Transition of purinergic signaling to drug discovery” [21].

To facilitate the targeting of neurons or other cells carrying P2X2Rs or P2Y_1_Rs in animal models of disorders of the CNS, we have generated transgenic BAC mice, which express these subunits as fluorescent fusion proteins (P2X2-TagRFP) or TagRFP as a reporter for P2Y_1_R expression, each under the control of their own promoters of the P2RX2 or P2RY1 gene, respectively. These animals should be used to (i) allow for a comprehensive microscopic mapping of the distribution of P2X2Rs and P2Y_1_Rs in mice and (ii) guide the identification of neurons for patch-clamp recordings or Ca^2+^ imaging experiments.

It should be noted here that in recent years, the methods for generation of transgenic mice have been developed very rapidly. In particular, the CRISPR/Cas technology has made targeted genetic modifications possible with the highest efficiency and in short order. However, the CRISPR/Cas technology was not available at the time when the present study was initiated. At that time, a large number of reports were available showing that BAC transgenic mice are valuable tools to map the tissue-specific gene expression pattern (for instance, see [22]). One of the best known projects is the Gene Expression Nervous System Atlas (GENSAT) project at the Rockefeller University (New York), which aims to map the expression of genes in the CNS of the mouse, using transgenic mouse (and in situ hybridization) techniques [23]. For these reasons, we have used the then established methods of BAC transgenesis*.*

## Material and methods

### Chemicals

Standard chemicals or products for immunohistochemistry were purchased from Sigma-Aldrich/Merck or Invitrogen/ThermoFisher, respectively.

### P2X receptor expression in *Xenopus laevis* oocytes

The cDNA encoding the mouse P2X2 subunit was a generous gift from T. Koshimizu (Faculty of Pharmaceutical Sciences, Kyoto University, Japan). After subcloning of the coding sequence into the oocyte expression vector pNKS affinity tag mutations (StrepII- and His_6_-tag) was introduced by site directed-mutagenesis following the QuikChange protocol (Stratagene, La Jolla, CA). The TagRFP coding sequence was cloned from the pTagRFP-N vector (Evrogen, Moscow, Russia) into the open reading frame by introduced HindIII and MfeI restriction sites to generate the mP2X2-StrepII-His-TagRFP oocyte expression construct. All constructs were verified by restriction analysis and nucleotide sequencing. Capped cRNAs were synthesized and injected into collagenase-defolliculated *X. laevis* oocytes using a Nanoliter 2000 injector (WPI, Sarasota, FL, USA) as described previously [24, 25]. Oocytes were cultured at 19 °C in sterile oocyte Ringer’s solution (ORi: 90 mM NaCl, 1 mM KCl, 1 mM CaCl_2_, 1 mM MgCl_2_, and 10 mM Hepes, pH 7.4) supplemented with 50 µg/ml gentamycin (Sigma-Aldrich, Taufkirchen, Germany).

### Protein labeling, purification, and PAGE

cRNA-injected oocytes were metabolically labeled by overnight incubation with l-[^35^S]methionine and, just before protein extraction additionally surface-labeled with Cy5-NHS ester (50 μg/ml, GE Healthcare Biosciences), an amine-reactive, membrane-impermeant fluorescent dye [24, 26]. His-tagged proteins were purified by Ni–NTA agarose (Qiagen, Hilden, Germany) chromatography from digitonin (1%, w/v) extracts of oocytes and analyzed by blue native PAGE (BN-PAGE; polyacrylamide gel electrophoresis) as detailed previously [24, 27]. If indicated, samples were treated before BN-PAGE for 1 h at 37 °C with 0.1% (w/v) sodium dodecyl sulfate (SDS) to induce partial dissociation of mP2X2 complexes. The BN-PAGE gels were destained before fluorescence imaging as detailed previously [28]. The Cy5-labeled plasma membrane-bound proteins and the TagRFP-tagged mP2X2 subunits were visualized by a Typhoon 9410 fluorescence scanner (GE Healthcare).

Figures were prepared by using ImageQuant TL v2005 (Amersham) for contrast adjustments, Adobe Photoshop CS 8.0 for level adjustment and cropping, and Microsoft PowerPoint 2000 for lettering.

### Cell cultures

All chemicals and media for cell culture were purchased from Invitrogen/ThermoFisher.

(i) *HEK cell cultures:* The propagation of human embryonic kidney cell line 293 (HEK293) was performed according to the American Type Culture Collection (ATCC) in Eagle’s minimal essential medium (MEM; Invitrogen, Carlsbad, USA) supplemented with 10% fetal bovine serum (FBS), 2 mM glutamine, 50 U/ml penicillin, and 50 µg/ml streptomycin in a humidified atmosphere with 5% CO_2_/95% air at 37 °C. Splitting of the cells was realized two or three times a week.
(ii) *Isolation and culture of mouse dorsal root ganglion cells:* The mice were killed under CO_2_, decapitated, and dorsal root ganglia (DRG) were removed aseptically from all levels of the spinal cord. After enzymatic and mechanical dissociation described previously [29], cells were cultured onto poly-L-lysin-coated glass coverslips in Dulbecco’s MEM containing 35 mM glucose, 2.5 mM L-glutamine, 15 mM HEPES, 50 µg/ml gentamicin, and 5% (FBS), supplemented with 10 µg/ml insulin, 5.5 µg/ml transferrin, and 5 ng/ml selenium. The cells were usually studied after 2–4 days in culture under 5% CO_2_ at 37 °C.
(iii) *Mixed astroglial-neuronal hippocampal cell cultures:* The preparation was performed as previously described [30]. In short, Wistar rat fetuses at gestational day 18 or from newborn rats at postnatal day 1 were used. The hippocampi were dissected, minced, and pooled in ice-cold (4 °C) HBSS (Life Technologies, Carlsbad, USA). Tissue samples were then dissociated in 1.5 ml of 0.05% trypsin (Invitrogen) in HBSS for 25 min at 37 °C. Enzymatic digestion was stopped by trypsin inhibitor (0.7 mg/ml; Sigma-Aldrich, Deisenhofen, Germany) added to a 1:1 mixture of Dulbecco’s modified Eagle medium (DMEM; Gibco, ThermoFisher Scientific Inc., USA) containing 36 mM D( +)-glucose, 15 mM HEPES (pH 7.4 with NaOH) plus 50 μg/ml gentamicin, and neurobasal medium (NBM; Gibco) mixed with B27 supplements (Gibco) containing 0.5 mM L-glutamine. After gentle trituration and centrifugation (80 × *g* for 5 min; repeated three times in total, separated by washout periods), the remaining cell pellet was re-suspended in the culture medium of the above composition. The final cell suspension was then seeded onto glass coverslips at a plating density of 4 × 103/cm^2^. Cultures were maintained in 2 ml of the culture medium in wells at 37 °C in a humidified atmosphere of 5% CO_2_ in air.

### Transient expression in HEK293 cells and patch-clamp electrophysiology

The mP2X2-StrepII-His coding sequence was subcloned from the pNKS vector into the pTagRFP-N vector by introduced HindIII/BamHI restriction sites. The resulting mP2X2-StrepII-His-TagRFP-pTagRFP-N plasmid was verified by restriction analysis and nucleotide sequencing. HEK293 cells were transfected by lipofectamine 2000 (Invitrogen) with the mP2X2-StrepII-His-TagRFP-pTagRFP-N plasmid. One or two days after transfection the P2X2R-mediated currents were analyzed by patch-clamp electrophysiology using 10 µM 2-meS-ATP as agonist.

### Generation of the transgenic P2X2 mice line C57BL/6 J-Tg(RP23-333M22P2X2-StrepHis-TagRFP)

#### Modification of the BAC clone and generation of the transgenic P2X2 mice line

Using the ENSEMBL database (http://www.ensembl.org), the BAC clone RP23-333M22 from the mouse C57BL/6 BAC library RPCI-23 [31] was identified to contain the entire P2RX2 gene. Bacterial artificial chromosome (BAC) clone RP23-333M22 was obtained from ImaGenes (Berlin, Germany) and verified by polymerase chain reaction (PCR) amplification and subsequent sequencing of exons 1–11 and flanking nucleotides. All BAC modifications were performed according to [32] (https://redrecombineering.ncifcrf.gov/), using vectors and bacterial lines freely obtainable from the National Cancer Institute in Frederick (NCI-Frederick, Bethesda, Maryland). A galK (galactokinase)-targeting cassette flanked by 500-bp arms that were homologous to the end of exon 11 of the P2X2 subunit gene was inserted into the BAC by homologous recombination. Next, the galK cassette was replaced by the DNA sequence for Strep-His-TagRFP as required. Positive BAC clones carrying the modification were identified using a SpeI restriction analysis, verified by PCR and DNA sequencing, linearized by the homing endonuclease PI-SceI, and dialyzed against the microinjection buffer (5 mM Tris, 0.1 mM EDTA, pH 7.6). The microinjection into pronuclei from C57BL/6 J oocytes was performed at the Max Planck Institute in Dresden as described previously [33]. Eight BAC transgenic founder mice were identified by PCR analysis for the presence of the TagRFP gene in genomic DNA from tail biopsies. Each founder line was maintained separately. The breeding of the C57BL/6 J-Tg(RP23-333M22P2X2-StrepHis-TagRFP) line was heterozygous with respect to the transgene. For all lines, both male and female offspring of the founder were analyzed.

#### DNA analysis

Genomic DNA from tail biopsies was extracted using REDExtract-N-Amp Kit (Sigma-Aldrich). The presence of the integrated BAC sequence was determined by PCR using the forward primer 5´-ACCAGCATATGGGACAAAGG-3´ and the reverse primer within the TagRFP sequence 5´-TGGGTGTGGTTGATGAAGG-3´, which amplify a 379-bp PCR product. Additionally, a PCR analysis of the unmodified part of exons 8 to 11 was included, to rate the DNA quality independently. PCR conditions were 94 °C for 30 s, 60 °C for 45 s, and 72 °C for 60 s, operating for 32 cycles.

### Generation of the transgenic P2Y_1_R mice line C57BL/6 J-Tg(RP23-452G4P2RY1-TagRFP)

#### Identification of a suitable BAC clone

Using the ENSEMBL database (http://www.ensembl.org), the BAC clone RP-23-452G4 from the mouse C57BL/6 BAC library RPCI-23 [31] was identified to contain the entire P2RY1 gene. According to this database, BAC clone 452G4 is 168, 946-bp long (base 60, 882, 308–61, 051, 254 of chromosome 3) and contains the entire P2RY1 gene (from base 61, 002, 795 to 61, 008, 979) with its protein-coding region from base 61, 003, 433 to 61, 004, 560 and an additional 120 kb and 42 kb of DNA flanking the 5´end of exon 1 and the 3´end of exon 2, respectively. The BAC clone 452G4 (in the pBACe3.6 vector) present in *E. coli* DH10B bacteria was purchased from ImaGenes (Berlin, Germany). According to the National Center for Biotechnology Information (NCBI) clone database, this BAC clone (CloneDB ID: 588,612) contained no other entire genes. The presence of the entire P2RY1 protein-coding sequence within BAC 452G4 was verified by polymerase chain reaction (PCR) and subsequent nucleotide sequencing analysis (MWG Biotech, Ebersberg, Germany) of exon 1.

#### Generation of the targeting cassette

A TagRFP-pA-FRT-PGK-gb2-Kan-FRT-cassette was generated via conventional and PCR-mediated cloning techniques using the pTagRFP-N vector (Evrogen; Moscow, Russia) and the FRT-PGK-gb2-Kan-FRT cassette of the Quick & Easy Conditional Knockout Kit (FRT/FLPe) (Gene Bridges GmbH; Heidelberg, Germany) as templates. The entire cassette was verified by nucleotide sequencing (MWG Biotech, Ebersberg, Germany).

For targeting the cassette to the region directly 5′ of the P2RY1 gene of the BAC clone 452G4, the verified TagRFP-pA-FRT-PGK-gb2-Kan-FRT-cassette was amplified by specifically designed PCR primers with 50 bp homology to the region directly 5′ of the start codon and the first 50 bp of the protein-coding sequence of the P2RY1 gene, respectively.

### Modification of the BAC clone and generation of the C57BL/6 J-Tg(RP23-452G4P2RY1-TagRFP) reporter mouse

Insertion of the amplified TagRFP-pA-FRT-PGK-gb2-Kan-FRT selection cassette directly 5′ of the start codon of the P2RY1 coding sequence and subsequent removal of the FRT-PGK-gb2-Kan-FRT cassette was performed via Red/ET and FLP-mediated homologous recombination, respectively, in *E. coli* DH10B cells containing the BAC clone 452G4 using the Quick & Easy Conditional Knockout Kit (FRT/FLPe) (Gene Bridges) according to the manufacturer’s protocol.

To verify proper recombination and to exclude the presence of major undesired recombination events after each round of homologous recombination, restriction analysis using the SpeI site present in the FRT-PGK-gb2-Kan-FRT cassette and 1% agarose gel electrophoresis was performed. At the end of the BAC modification process, the modified region of the BAC clone was verified by nucleotide sequencing.

For pronuclear injections, BAC DNA was purified by the BAC 100 Kit according to the manufacturer’s protocol (Macherey–Nagel, Düren, Germany). The concentration of the isolated BAC DNA was determined with the Nanodrop 1000 spectrometer (ThermoFisher Scientific, USA), and 20 µg BAC DNA was linearized via the PI-SceI site present in the pBACe3.6 vector by the homing endonuclease PI-SceI (New England Biolabs, Ipswich, USA). The linear BAC DNA was dialyzed overnight against injection buffer (5 mM Tris/HCl, 0.1 mM EDTA, pH 7.6) and verified by sequence analysis (MWG Biotech).

Pronuclear injection of the modified BAC DNA (3 ng/l) into fertilized oocytes of C57BL/6 J mice, implantation into pseudopregnant C57BL/6 J females, and breeding of the founder mice were performed at the transgenic core facility of the Max Planck Institute of Molecular Cell Biology and Genetics (MPI-CBG) (Dresden, Germany) as described previously [33]. PCR analysis for the presence of TagRFP revealed nine transgenic founder mice, of which eight showed germline transmission of the transgene. The breeding of the C57BL/6 J-Tg(RP23-452G4P2RY1-TagRFP) line was heterozygous with respect to the transgene.

### DNA analysis

Genomic DNA from tail biopsies was extracted using REDExtract-N-Amp Kit (Sigma-Aldrich). The presence of the integrated BAC sequence was determined by via multiplex-PCR with the following primer pairs, amplifying a 460-bp DNA fragment of the transgene (5′-TGG GTG TGG TTG ATG AAG G-3′ and 5′-ACC AGC ATA TGG GAC AAA GG-3′) and a 1 kb fragment of the actin beta gene as an internal control (5′-GAT GAC GAT ATC GCT GCG CTG GTC G-3′ and 5′-GCC TGT GGT ACG ACC AGA GGC ATA CAG-3′). PCR conditions were 94 °C for 30 s, 60 °C for 45 s, and 72 °C for 60 s, operating for 32 cycles.

### Mice breeding (both mice lines)

C57BL/6 J mice were bred at the Institute of Laboratory Animal Science of the RWTH Aachen and at the animal house of the Rudolf Boehm Institute of Pharmacology and Toxicology, University of Leipzig (both, Germany). All animals were kept at a standard 12 h light–dark cycle and supplied with food and water ad libitum under defined pathogen-free conditions. All transgenic lines were bred heterozygous.

Animal experiments were in compliance with the European Union legislation (Directive 86/609/EEC), approved by the Committee of Animal Care and Use of the relevant local governmental body and in accordance with the European Federation of European Laboratory Animal Science Associations (FELASA) standards and were approved by the relevant regulatory authorities (Leipzig: T36/12; T21/13; T05/16; Aachen: *Xenopus laevis* oocytes for heterologous protein expression: 9.93.2.10.44.07.104; mice: 10,857 A4).

### Visualization/immunolabeling/cell imaging of transgenic and control mice

#### Immunolabeling

(i) *Living cells*
  For labeling of living cells, cultures (e.g., DRG neurons of P2X2 mouse line, see (ii)) were washed twice with PBS (pH 7.4) at room temperature. Unspecific binding was blocked with 10% FBS in HBSS at 37 °C for 30 min. To identify non-peptidergic neurons in the culture 30 min incubation with 1 µg/ml biotinylated *Griffonia simplicifolia* isolectin-B4 (GSI-B4; Sigma) was performed at 37 °C. Cells were washed three times with PBS, before carbocyanine Cy5-labeled streptavidin was applied for 30 min at 37 °C in HBSS with 10% fetal calf serum (FCS), respectively. After washing the cells with PBS at room temperature, 1 µg/ml Hoechst 33342 in PBS for 5 min was used to visualize the nuclei. PBS was replaced by HEPES buffered solution (HBS, pH 7.4) containing 155 mM NaCl, 5 mM KCl, 2 mM CaCl_2_, 2 mM MgCl_2_, 10 mM glucose, and 10 mM HEPES for imaging.
(ii) *DRG primary cultures*
  Cells were fixed with a 2% paraformaldehyde (PFA) solution in PBS (pH 7.4) for 10 min at 4 °C and washed twice with PBS. Unspecific binding sites were blocked for 30 min by 10% fetal goat serum and 1% bovine serum albumin (BSA) in PBS with 0.05% Triton X100, to permeabilize the cell membranes. To enhance the signal of the fluorescence protein, a polyclonal rabbit antibody against TagRFP (Evrogen, Moscow, Russia) was used in combination with Cy2-labeled goat anti-rabbit IgG (Jackson ImmunoResearch Inc., PA, USA).
(iii) *Mixed astroglial-neuronal hippocampal cell cultures* (*of *P2RY1-TagRFP mice)
  At DIV 8, a scratch lesion diagonally through the mixed hippocampal culture using a pipette tip was performed. At DIV 10, the cultures were washed with PBS and then treated with ice-cold 2% PFA in PBS (pH 7.4) for 10 min at 4 °C and washed twice with PBS.
  Unspecific binding sites were blocked for 30 min by blocking buffer (10% FBS and 1% BSA in PBS with 0.05% Triton X100) at room temperature. For the immunolabeling procedure, cells were incubated with the primary antibody (mouse anti-GFAP, 1:1000, Sigma-Aldrich) overnight at 4 °C. After washing in PBS, subsequent incubation with secondary antibody (donkey anti-rabbit IgG conjugated to Cy2, 1:500; Jackson ImmunoResearch) followed for 2 h at room temperature. Hoechst 33342 (Hoe, 10 µg/ml; Molecular Probes, Leiden, Netherlands) was added for 5 min before the end of this incubation period.
(iv) *Slices/sections from mice*
  The mouse tissues of young and adult mice were fixated by transcardial perfusion under thiopental sodium anesthesia with 4% PFA solution in PBS (pH 7.4). After removal of the brain, the subsequent postfixation for 1 h was performed in the same solution.

Different kinds of tissue slices were used: (a) cryosections and (b) free-floating slices.

- Cryo slices (18–20 µm): The tissues were cryoprotected in 30% sucrose in PBS (pH 7.4). Coronal sections were sliced with a cryostat (Leica, CM3050S, Wetzlar, Germany) using tissue freezing medium von Jung (Leica Instruments GmbH, Nussloch, Germany). The cryoprotectant was removed by three wash steps in PBS, followed by postfixation with cold methanol. Tyramide signal amplification (TSA) used for P2X2R mouse line): Therefore, endogenous peroxidases were blocked with 0.3% H_2_O_2_. Ten percent non-fat dry milk in PBS (pH 7.4) with 0.2% Triton X100 and avidin blocking solution (Avidin/Biotin Blocking, Vector Labs, Burlingame, USA) was used to saturate non-specific binding sites. Followed incubation with primary antibody against TagRFP was performed in PBS solution with 0.2% Triton X100 with 1% normal goat serum (NGS) and biotin blocking solution. Biotin was added in a next step by biotin-conjugated goat anti-rabbit IgG (Jackson ImmunoResearch Inc., PA, USA). After coupling of streptavidin-HRP (NEN Life Science Products Inc., Boston, USA), biotin-conjugated tyramide solution (NEN Life Science Products Inc., Boston, USA) was deployed prior incubation with Cy5-labeled streptavidin.
- Free-floating slices (50 µm): After pericardial perfusion fixation, the brains were cut into 50 µm slices using a vibratome (Leica, Typ VT 1200S, Nussloch, Germany) and collected and stored at 4 °C in PBS.

For the immunolabeling procedure, slices were rinsed three times with PBS for 5 min to remove the cryoprotectant compound and were subsequently incubated with a blocking buffer (10% NGS, 0.2% BSA in PBS) for 60 min at room temperature. After the incubation with primary antibodies overnight at 4 °C, subsequent incubation with secondary antibodies followed for 2 h at room temperature.

For expression analysis and cell identification, the following antisera were used: polyclonal rabbit anti-TagRFP (AB233, dilution 1:2000; Evrogen, Moscow, Russia), monoclonal mouse anti-microtubule associated protein-2 (MAP2) (MAB3418, dilution 1:1000; Merck Millipore, Darmstadt, Germany), monoclonal mouse anti-neuron-specific nuclear protein (NeuN) (MAB377, dilution 1:2000, Merck Millipore, Darmstadt, Germany), and monoclonal mouse anti-glial fibrillary acidic protein (GFAP) (WH0002670M1 dilution 1:1000; Sigma-Aldrich).

For primary antibody detection polyclonal goat or donkey anti-rabbit IgG conjugated to Cy2 (dilution 1:500; Jackson ImmunoResearch) and goat or donkey anti-mouse IgG conjugated to Cy5 (dilution 1:500; Jackson ImmunoResearch) were used. For nuclear staining, Hoechst 33342 (Hoe, 10 µg/ml; Molecular Probes) or DAPI (DAPI hydrochloride 300 nM, Invitrogen FluoroPure grade, ThermoFisher Scientific Inc.) was added for 5 min, and residual dye was removed by a final PBS washing step. After staining procedure, the slices were mounted onto glass slides prior to imaging. When slices were incubated in PBS without the primary antibody, or with primary antibody which had been pre-absorbed with peptide antigen for 1 h before use, no immunofluorescence was observed.

All stained sections were dehydrated in a series of graded ethanol, processed through n-butylacetate and coverslipped with Entellan (Merck, Darmstadt, Germany) or RotiMount FluorCare (Roth, Karlsruhe, Germany).

### Confocal microscopy/fluorescence microscopy

All slides were visualized by using a (i) Zeiss Axiovert 200 M fluorescence microscope equipped with an HBO 150 lamp or (ii) by a confocal laser scanning microscope (cLSM 510 Meta, Zeiss, Oberkochen, Germany) using excitation wavelengths of 488 nm (argon, yellow-green Cy2/Alexa 488-immunofluorescence labeling), 543 nm (helium/neon1, red fluorescence), and 633 nm (helium/neon2, blue Cy5/DyLight-labeling). An ultraviolet laser (362 nm) was used to illustrate the blue-cyan Hoechst 33342 auto-fluorescence. Color coding of the Cy5-immunofluorescence to allow optimal illustration of all labeled structures was performed.

## Results

### P2X2R transgenic mouse model

To facilitate the morphological identification and functional characterization of cells expressing the P2X2R, we generated C57BL/6 J-Tg(RP23-333M22P2X2-StrepHis-TagRFP) BAC-transgenic mice expressing the P2X2R protein fused to TagRFP under control of the regulatory elements of the P2RX2 gene.

We used the red fluorescence protein TagRFP, a monomeric derivative of eqFP578 [34], which is characterized by high brightness, complete chromophore maturation, prolonged fluorescence lifetime, and high pH-stability [35]. These properties make TagRFP to an excellent tag for protein localization studies and fluorescence resonance energy transfer applications.

**Fig. 1:**
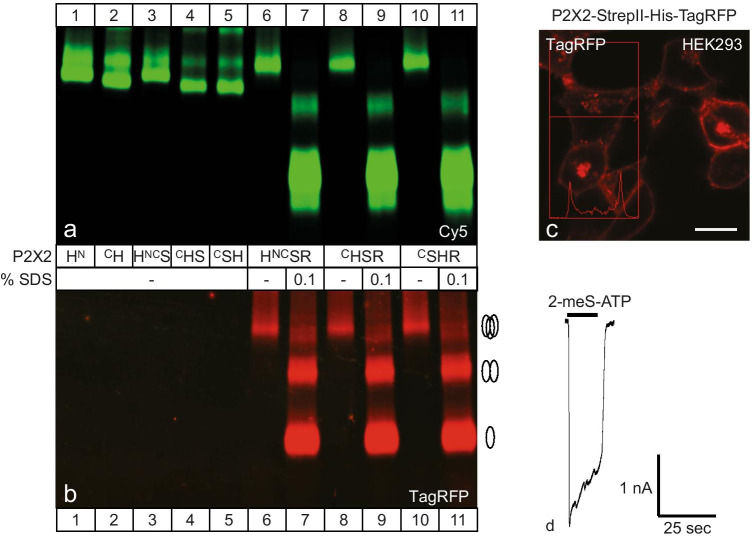
Plasma membrane expression, oligomeric assembly, and function of affinity- and TagRFP-tagged mP2X2 receptors. **a**, **b** For biochemical analysis, *X. laevis* oocytes expressing the indicated mP2X2 subunit tagged at the N-terminus (^N^) or C-terminus (^C^) with a His-(H), StrepII (S), or TagRFP-tag (R) were surface labeled with the membrane-impermeant fluorescent Cy5 dye before protein purification. The indicated proteins were purified under non-denaturing conditions from *X. laevis* oocytes by Ni^2+^-NTA chromatography, resolved by BN-PAGE (4–20%) in its non-denatured (native, no SDS) or partially denatured (0.1% SDS) state and visualized by typhoon fluorescence scanning. **a** Cy5-labeled surface form of the indicated mP2X2 proteins in their native or partial SDS denatured forms as indicated. **b** TagRFP fluorescence of the BN-PAGE gel shown in **a**. The number of protomers included in the respective bands is schematically indicated at the right margin. **c**, **d** Subcellular distribution and function of mouse P2X2-StrepII-His-RFP receptor construct transiently expressed in HEK293 cells. **c** Confocal laser scanning microscopy shows plasma membrane bound TagRFR fluorescence, which is also highlighted by the fluorescence intensity diagram along a cross section through the cell. **d** Representative current trace recorded by the whole-cell patch-clamp technique of a HEK293 cell transiently expressing the mouse P2X2-StrepII-His-RFP receptor construct. The horizontal black bar indicates the duration of application of the agonist 2-meS-ATP (10 µM). Scale bar: c = 10 µm

For the generation of the C57BL/6 J-Tg(RP23-333M22P2X2-StrepHis-TagRFP) transgenic mouse model, which expresses a fusion protein consisting of the mP2X2R, affinity tags, and TagRFP, extensive preliminary studies were necessary: It was crucial to ensure that the fusion of the affinity tags (His_6_- and StrepII-tag) as well as the TagRFP would not affect protein expression, trimeric assembly, function, and plasma membrane expression. As an example for this studies, Fig. 1a and b show different constructs of the mP2X2R with N- or C-terminal fused affinity tags and C-terminal fused TagRFP expressed in *Xenopus laevis* oocytes, purified in non-denatured form by Ni^2+^-affinity chromatography, and separated by Blue-native gel electrophoreses (BN-PAGE) in their native forms. After staining of the intact oocytes with the membrane-impermeant Cy5 dye, the plasma membrane fraction of the indicated constructs is shown by Cy5 fluorescence (Fig. 1a). The red fluorescence of TagRFP of the corresponding fused constructs is shown (Fig. 1b). All TagRFP fused mP2X2R constructs were found to be expressed at the plasma membrane and were present in their native/non-denatured form as trimers.

The mP2X2-StrepII-His-TagRFP construct is transiently expressed in HEK293 cells and shows a plasma membrane-bound TagRFP fluorescence as well (Fig. 1c). Figure 1d displays a representative current trace of a HEK293 cell expressing the mP2X2-StrepII-His-TagRFP of a whole-cell measurement by patch-clamp electrophysiology in which the mP2X2R-mediated current response was induced by 2-meS-ATP, demonstrating that the mP2X2-StrepII-His-TagRFP receptor is functional.

**Fig. 2:**
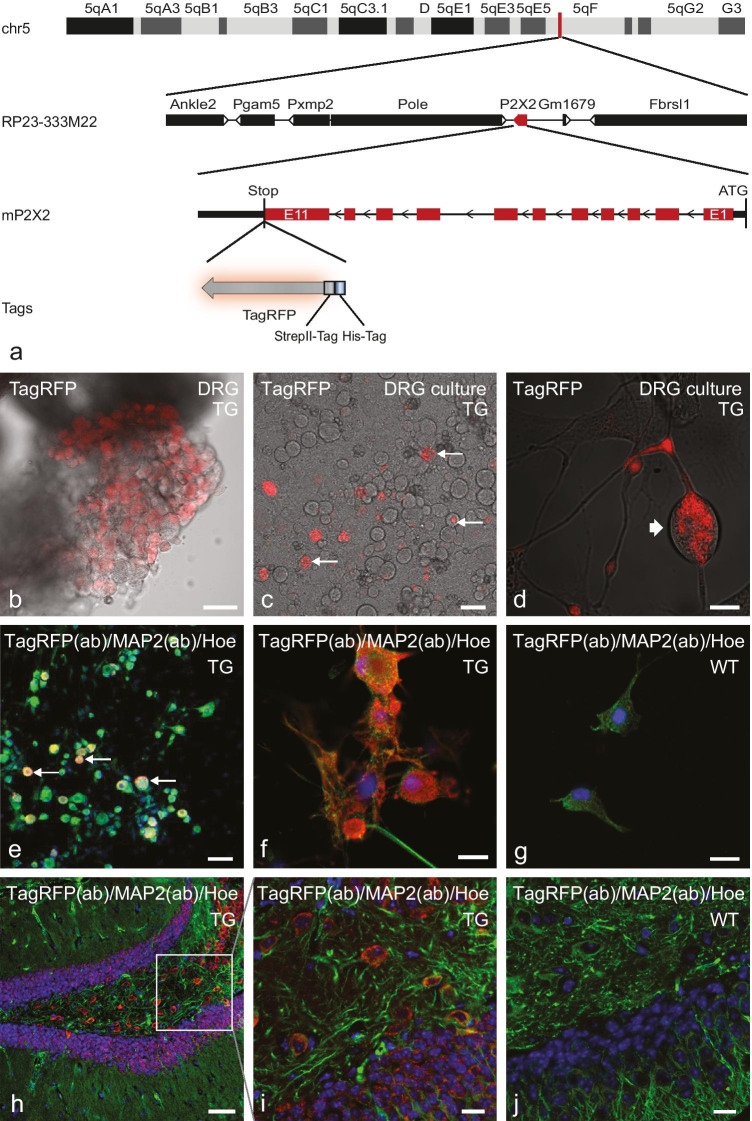
Transgenic P2X2 mice line C57BL/6 J-Tg(RP23-333M22P2X2-StrepHis-TagRFP). **a** Schematic illustration of a region of the mouse chromosome 5 indicating the position of the origin of BAC clone RP23-333M22 (vertical red line). The BAC clone RP23-333M22 containing the full-length P2RX2 gene (red box) is shown below. The P2RX2 gene is shown with its 11 exons. The StrepII-His-TagRFP encoding sequence (StrepII-His-TagRFP cassette) was inserted directly upstream of the stop codon (exon 11). **b–j** Confocal laser scanning microscopy of TagRFP fluorescence and TagRFP-/neuronal co-immunostaining. **b–d** show an overlay of confocal TagRFP fluorescence imaging and transmitted light imaging. Images of a **b** native dorsal root ganglion, **c–g** cultured DRG neurons **c, d** native; **e–g** PFA fixed), and **h–i** PFA-fixed cryosections of the dentate gyrus/CA4 region of the hippocampus of an adult C57BL/6 J-Tg(RP23-333M22P2X2-StrepHis-TagRFP) mouse. **b** Native TagRFP-fluorescence (red) of a crush preparation of a dorsal root ganglion. **c**, **d** Native TagRFP-fluorescence (red, arrows) of cultured DRG neurons (2 days in vitro (DIV 2)). **e**, **f** TagRFP and MAP2 co-immunostaining (overlay), TagRFP red, MAP2 green; antibodies (ab)) of PFA-fixed cultured DRG neurons (DIV 2). Only a fraction of MAP2-positive DRG neurons do co-express the P2X2-StrepHis-TagRFP transgene (arrows). **g** TagRFP and MAP2 co-immunostaining (overlay, TagRFP red, MAP2 green) of PFA-fixed cultured DRG neurons of a wild-type C57BL/6 J mouse (littermate control). No TagRFP staining was found. **h–j** TagRFP and MAP2 co-immunostaining (overlay, TagRFP red, MAP2 green) of PFA-fixed cryosections of the dentate gyrus/CA4 region of the hippocampus. **h**, **i** C57BL/6 J-Tg(RP23-333M22P2X2-StrepHis-TagRFP) mouse (adult), **j** wild-type C57BL/6 J mouse (littermate control). Scale bars: b, c, e, h = 50 µm; d, f, g, i, j = 10 µm

Figure 2a shows the schematic illustration of the Tg(RP23-333M22P2X2-StrepHis-TagRFP) transgene, which drives the P2X2-TagRFP expression under control of the promoter of the P2RX2 gene. In the C57BL/6 J-Tg(RP23-333M22P2X2-StrepHis-TagRFP) BAC-transgenic mice, we were able to detect TagRFP fluorescence in dorsal root ganglia (DRG) neurons, and we could visualize the receptor in cerebral structures, including hippocampal tissues. After pronuclear injection of the modified BAC Tg(RP23-333M22P2X2-StrepHis-TagRFP), eight founder lines were obtained, and offspring were analyzed regarding the TagRFP fluorescence within the hippocampus. Only one line shows a strong fluorescence intensity and is used for further breeding (Suppl. Figure 1; line G503 was chosen for further breeding).

Expression of P2X2 fusion receptor is demonstrated in a large number of cells of an entire DRG (squeeze preparation) of hemizygote transgenic mice by native TagRFP fluorescence (Fig. 2b). In cultured DRG neurons, a small number of cells only (small arrows) exhibit fluorescent receptors, most pronounced after 2 days in culture (Fig. 2c). The localization of the receptor at this stage of cultivation shows predominantly in intracellular membranes (Fig. 2d; thick arrow) and appears infrequently on the cell surface, indicating an active regulation of surface expression. Co-immunolabeling with antibodies against TagRFP and against the neuronal markerMAP2 confirms the expression in neurons (Fig. 2e, f), whereas no TagRFP fluorescence could be detected in littermate controls (WT) (Fig. 2g). In tissue sections, we could observe the expression of the fusion-receptor only by signal amplification, taking advantage of antibodies specific for TagRFP. This prevented the tracking of living cells in tissue sections of P2X2R-TagRFP mice by native TagRFP fluorescence. Using TagRFP-immunostaining, P2X2-TagRFP expression is found in neurons in the dentate gyrus within the hippocampus (Fig. 2h, i) of transgenic (TG) mice, but not in littermate controls (WT) (Fig. 2j).

**Fig. 3:**
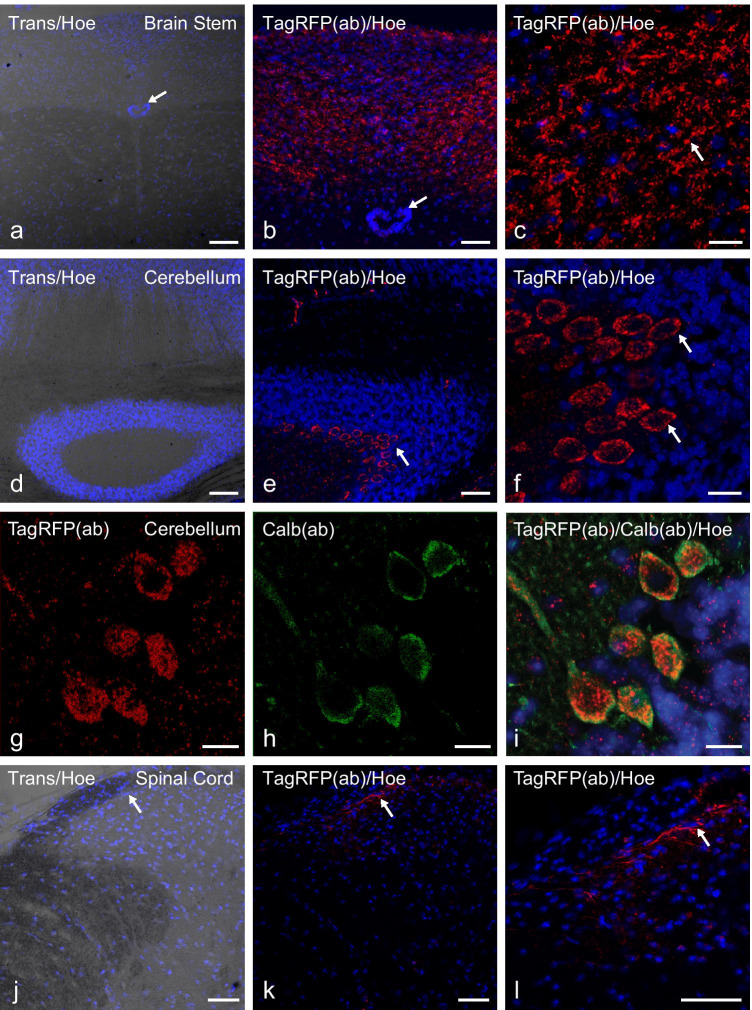
CNS Expression of P2X2-TagRFP in transgenic P2X2 mice. Confocal imaging of TagRFP immunofluorescence (red) and Hoechst staining (blue) of PFA-fixed cryosections of the indicated CNS regions of the transgenic P2X2 mice line C57BL/6 J-Tg(RP23-333M22P2X2-StrepHis-TagRFP). TagRFP immunofluorescence and Hoechst staining in coronal sections of the medulla within the brain stem **a**, **b** and in fibers within the nucleus tractus solitarius (NTS) **c** of the cerebellum **d–f** and **g–i** and the spinal cord **j–l**. **a, d, j** Overlay of Hoechst staining and transmitted light imaging. **b, c** and **e, f** and **k, l** Overlay fluorescence imaging of TagRFP immunofluorescence and Hoechst staining in different magnifications. The arrows in **a, b** or **c** show the central canal or TagRFP immunofluorescence in fibers within the NTS, respectively. The arrows in **e, f** or **j–l** highlight the plasma membrane bound TagRFP staining in Purkinje neurons or the small fraction of TagRFP staining within superficial dorsal horn laminae of the spinal cord, respectively. **g–i** immunofluorescence of TagRFP (red) and calbindin (green) and Hoechst staining in the cerebellum. **g** Overlay of TagRFP immunofluorescence and Hoechst staining. **h** Overlay of calbindin immunofluorescence and Hoechst staining. **i** Overlay of calbindin and TagRFP immunofluorescence and Hoechst staining. Scale bars: a, d = 100 μm; b, e, j, k, l = 50 μm; c, f, g, h, i = 20 µm

Furthermore, we found P2X2-TagRFP expression in the brain stem (Fig. 3a–c) in the medulla in the area postrema as well as in fibers within the nucleus tractus solitarius, in the cerebellum (Fig. 3d–f), where most calbindin-positive cells expressed P2X2-TagRFP and in the superficial laminae of the dorsal horn in the spinal cord (Fig. 3g–i).

### P2Y_1_R transgenic mouse model

**Fig. 4:**
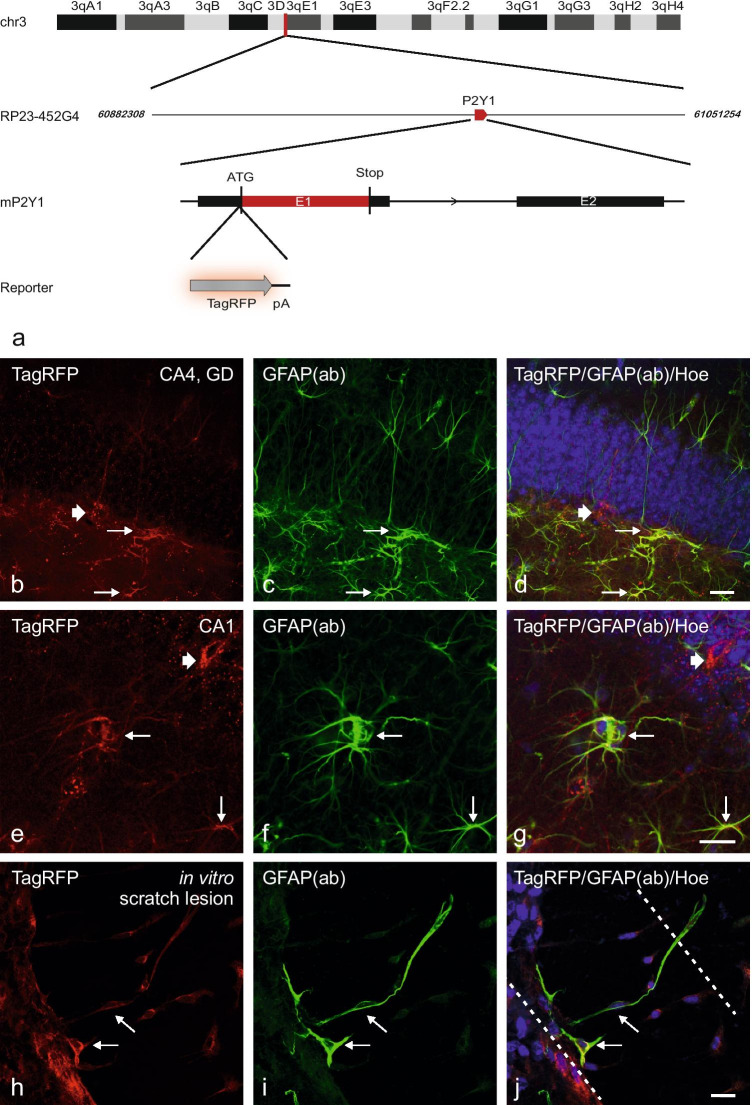
Transgenic P2Y_1_R mice line C57BL/6 J-Tg(RP23-452G4P2RY1-TagRFP). **a** Schematic illustration of a region of the mouse chromosome 3 indicating the position of the origin of BAC clone RP23-452G4 (vertical red line); BAC clone RP23-452G4 containing the full-length P2RY1 gene (red box) is shown below. The P2RY1 gene includes two exons. Exon 1 (E1) contains the entire protein-coding sequence of the P2Y_1_R. The TagRFP-pA encoding sequence (TagRFP-pA cassette) was inserted directly upstream of the P2RY1 translation start site (ATG). **b–g** cLSM imaging of TagRFP fluorescence and astroglial co-immunostaining. Images of PFA-fixed cryosections of the dentate gyrus (GD)/CA4 region of the hippocampus of an adult C57BL/6 J-Tg(RP23-452G4P2RY1-TagRFP) mouse. **b, d, e, g** Native TagRFP-fluorescence (red). **c, f** Immunostaining with the astrocyte marker GFAP (antibody, ab) revealed that GFAP co-localizes with TagRFP, indicating that astrocytes (some are exemplarily marked by thin arrows) express the P2Y_1_R. Furthermore, also GFAP negative neurons (thick arrow in b, d, e, g) express the P2Y_1_R within the hippocampus as well. **d, g** Overlay of native TagRFP fluorescence, GFAP and Hoechst staining. **h, i** Scratch lesion in a neuron-glia mixed hippocampal cell culture (DIV 10). Four days after lesion outgrowing (or newly formed) TagRFP-positive and GFAP-positive astrocytes were observed in the injury area (arrows). Scale bars: b**–**j = 20 µm

We also generated C57BL/6 J-Tg(RP23-452G4P2RY1-TagRFP) BAC-transgenic mice-expressing TagRFP under control of the regulatory elements of the P2RY1 gene to advance the morphological identification and functional characterization of P2Y_1_R-expressing cells. Figure 4a shows the schematic illustration of the Tg(RP23-452G4P2RY1-TagRFP) transgene, which drives the TagRFP expression under control of the promoter of the P2RY1 gene. After pronuclear injection of the modified BAC Tg(RP23-452G4P2RY1-TagRFP), eight fertile founder lines were obtained, and offspring were analyzed regarding the TagRFP fluorescence within the hippocampus. The hippocampus was selected as a particularly suitable region for a screening analysis of different founder lines, since the expression of the P2Y_1_R in this CNS region has been repeatedly demonstrated in various studies (for instance, see [36, 37]). However, great differences appeared in the intensity of native TagRFP fluorescence in different founder lines and their offspring. Five lines show viable native TagRFP fluorescence, and one of them shows particularly strong fluorescence intensity and is chosen for further breeding and characterization (Suppl. Figure 2; line T933 was chosen for further breeding). Please note that the expression pattern is conserved among the different founder lines that showed native TagRFP fluorescence (Suppl. Figure 2c, e, f, g, h), which suggests that the TagRFP expression is independent from the position of integration within the genome.

**Fig. 5:**
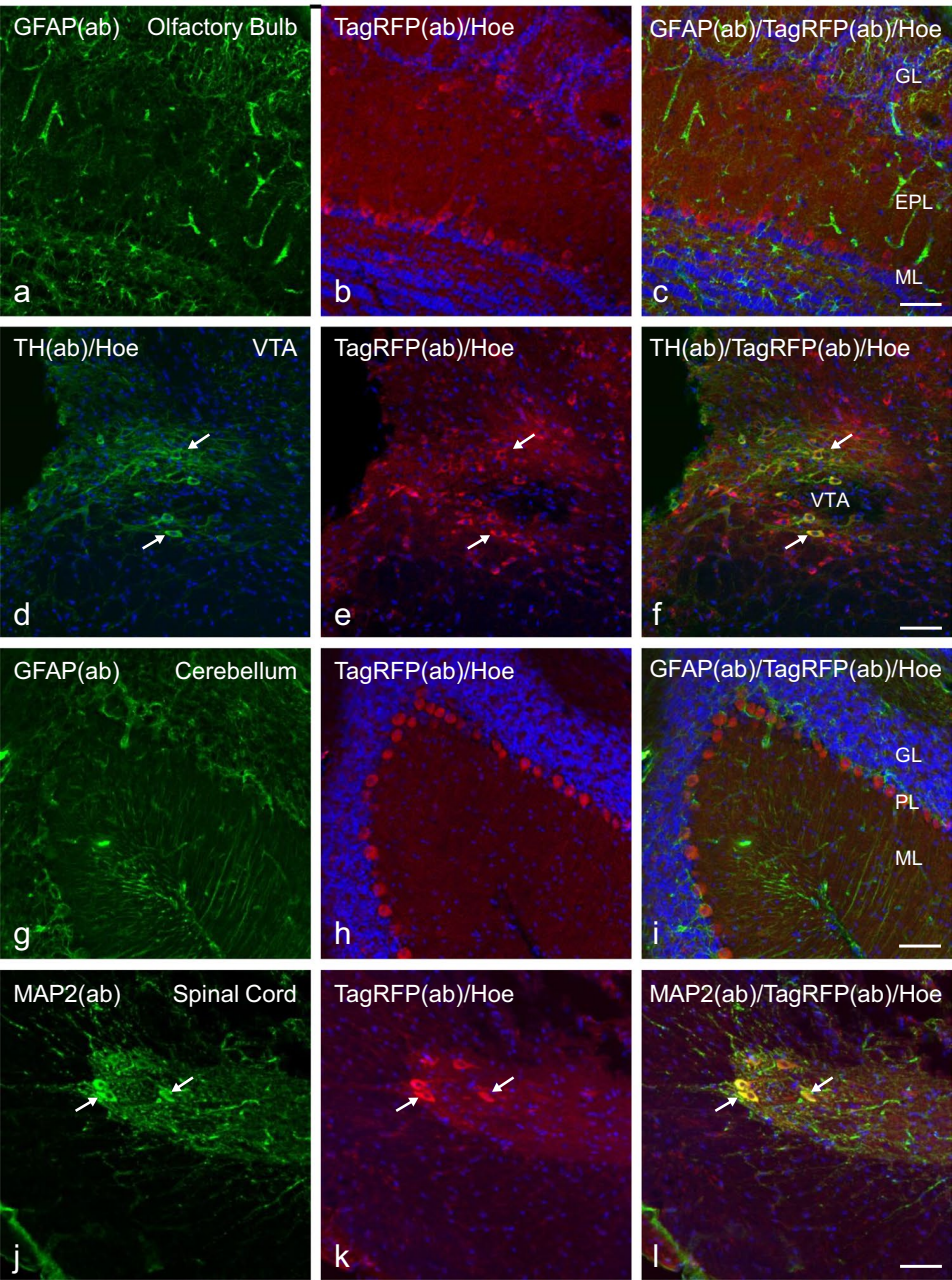
CNS Expression of TagRFP in transgenic P2Y1 reporter mice. Confocal imaging of TagRFP immunofluorescence (red), Hoechst staining (blue) and GFAP, TH or MAP2 co-immunofluorescence (green) as indicated of PFA-fixed cryosections of the indicated CNS regions of the transgenic P2Y_1_R mice line C57BL/6 J-Tg(RP23-452G4P2RY1-TagRFP). TagRFP immunofluorescence and Hoechst staining in sections of the olfactory bulb **a–c**, the VTA **d–f**, the cerebellum **g–i**, and the spinal cord **j–l**. **a–c** show TagRFP immunofluorescence in neurons of the mitral cell (ML) layer and at the border between the external plexiform layer (EPL) and the glomerular layer (GL) of the olfactory bulb, but no co-localization with GFAP co-immunofluorescence **a, c**. **d–f** TagRFP immunofluorescence in tyrosine hydroxylase (TH)-positive dopaminergic neurons (arrows) and also a fraction of TH-negative neurons of the VTA. **g–i** TagRFP immunofluorescence in Purkinje neurons of the cerebellum. GFAP co-immunofluorescence **g, i** shows no co-localization with TagRFP **a,c**. **j–l** TagRFP immunofluorescence in MAP2-positive neurons of laminae I-II, and with less intensity in laminae III-V of the dorsal horn of the spinal cord (Th3). The arrows show neurons with strong MAP2/TagRFP co-staining. Scale bars: c, f, i, l = 50 µm

In these P2Y_1_R BAC-transgenic reporter mice, we were able to detect TagRFP fluorescence in several cerebral tissues, including hippocampal neurons and astrocytes. We examined the expression levels of native TagRFP fluorescence in P2Y_1_R-expressing neurons and astrocytes (Fig. 4b–j) and also enhanced the visibility of TagRFP-expressing cells by using TagRFP-specific immunocytochemistry (Fig. 5a–l).

**Fig. 6:**
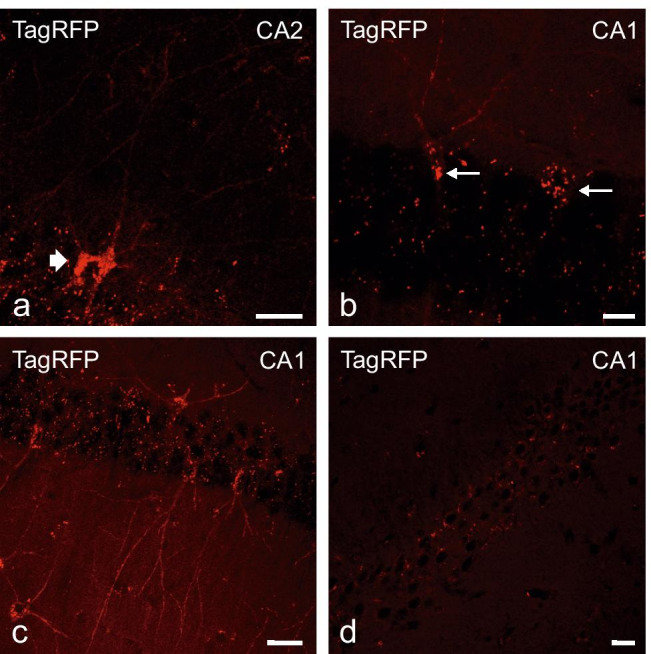
Changes of TagRFP fluorescence during breeding of the transgenic P2Y_1_R mice line C57BL/6 J-Tg(RP23-452G4P2RY1-TagRFP). Screening of native TagRFP fluorescence in hippocampal cryosections (CA1/2 as indicated) of different generations of the transgenic P2Y_1_R mice line C57BL/6 J-Tg(RP23-452G4P2RY1-TagRFP). **a** Representative strong native TagRFP-fluorescence in neurons (cell body, cell processes) of first generations (first year of breeding) of C57BL/6 J-Tg(RP23-452G4P2RY1-TagRFP) mice. **b, c** Representative native TagRFP-fluorescence in later generations (> 1.5 years of breeding) of C57BL/6 J-Tg(RP23-452G4P2RY1-TagRFP) adult mice. TagRFP fluorescence appears in form of granules in the neuronal cell bodies (arrows). Additionally, native TagRFP-fluorescence-positive processes were still observed. **d** Finally, after > 2 years of breeding, characteristic cellular native TagRFP-fluorescence disappeared. Only some accumulation of granules in the cell bodies was still found. Scale bars: a, c, d = 20 µm; b = 10 µm

As shown in Fig. 4, native TagRFP fluorescence is found in cells within the hippocampus, i.e., without the need of antibody-dependent signal enhancement. Figure 4b, d, e, and g show TagRFP fluorescence in the soma and the entire processes of hippocampal neurons (thick arrows) located in the dentate gyrus and CA1/2 regions at the border of the molecular and polymorphic layer to the granular layer of the dentate gyrus (e.g., Fig. 4b–d), suggesting that the TagRFP reporter protein may allow for tracking the projections of P2Y_1_R-expressing neurons (see also Fig. 6a–c). Additionally, also the TagRFP expression on GFAP-positive astrocytes is observed (Fig. 4b–g, thin arrows). Some TagRFP-positive cells could be identified as neurons based on co-localization with multiple neuronal markers. In an additional analysis, an in vitro lesion model for mechanical injury was used [38]. We observe the outgrowth of TagRFP- and GFAP-co-expressing astrocytes in the lesion area in hippocampal neuron-glia mixed cell cultures after a scratch lesion (Fig. 4h–j). These astrocytes develop fibers in the lesion area, and also the presence of completely (cell body, processes) double-labeled cells is observed (Fig. 4h–j, thin arrows). This indicates a strong P2Y_1_R expression during astroglial proliferation or migration processes and thus may support the findings of previous studies about a role of P2Y_1_Rs in proliferation and regeneration [12–16].

In a next step of our investigations, we extended our P2Y_1_R expression analysis to further brain regions. TagRFP immunostaining and hence P2RY1 expression are detected in (i) the mitral cell layer and at the border between the external plexiform layer and the glomerular layer of the olfactory bulb, indicating P2Y_1_R expression in mitral and external tufted cells (Fig. 5a–c); (ii) the ventral tegmental area (VTA) (Fig. 5d–f); (iii) the Purkinje cell layer of the cerebellum (Fig. 5g–i); and (iv) the laminae I-II and with less intensity in laminae III-V of the dorsal horn of the spinal cord (Fig. 5j–l). In the VTA, the majority of the TagRFP reporter expressing cells is identified as dopaminergic neurons as revealed by co-staining with an antibody against tyrosine hydroxylase (Fig. 5d–f, examples are indicated by arrows).

### Loss of fluorescence over time 

At the beginning of the P2Y_1_R project, different transgenic founder lines were investigated and analyzed with respect to expression in the hippocampus. As detailed above, a comparable qualitative expression pattern in glial cells and neurons could be demonstrated within the different lines (offspring of different founder lines, c.f. Suppl. Figure 2), but the intensity of fluorescence differed between the lines. The line with the strongest expression (in terms of the strongest TagRFP fluorescence intensity) was selected for further breeding, but an intermittently occurring low fluorescence was observed in some transgenics during breeding. In the further course of breeding, the initially quite strong fluorescence intensity decreased from generation to generation. This is illustrated in Fig. 6. While in the first generations a pronounced TagRFP fluorescence in cell bodies and processes of hippocampal neurons were detected (Fig. 6a), the fluorescence became successively weaker in the course of further generations (Fig. 6b, c) until after about 2 years of breeding; no fluorescence is detectable in neuronal processes; and also the cell bodies show only a weak residual labeling (Fig. 6d). It was also observed that over time, the fluorescence became increasingly granular in the cell bodies.

## Discussion

### Transgenic mice as tools to analyze the P2R expression

#### Our transgenic models

In the studies described here, we have generated (i) C57BL/6 J BAC transgenic mice that express the P2X2R subunits as fluorescent fusion protein (P2X2-TagRFP) controlled by its own promoter and (ii) a BAC P2Y_1_R TagRFP reporter mouse expressing a TagRFP reporter for the P2RY1 gene expression to identify cells in situ carrying P2X2Rs or expressing the P2YR1 gene, respectively. We show that the targeted receptors are expressed in different cells and tissues of the transgenic mice lines. In our C57BL/6 J BAC transgenic mice, we were able to demonstrate expression of the P2X2R in a subset of DRG neurons, the brain stem, the hippocampus, as well as on Purkinje neurons of the cerebellum. The expression pattern on DRG neurons and in the specified locations of the CNS, such as the hippocampus, was recently confirmed by other P2X2R transgenic mouse lines detailed below [39, 40]. However, it should be reemphasized here that the weak fluorescence intensity in our P2X2R-TagRFP mouse and the therefore necessary use of anti-TagRFP immunostaining and subsequent signal amplification have precluded tracking of living cells or identifying them in sections for targeted examination, e.g., by patch-clamp electrophysiology.

Our P2Y_1_R reporter mice confirmed the widespread expression of the P2RY1 gene in the CNS that was previously indicated by RT-PCR, in situ hybridization and immunocytochemistry [e.g. 12–15, 37, 41–43]. However, TagRFP reporter expression indicates for the first time P2RY1 gene expression and thus P2Y_1_R expression in mouse Purkinje cells, which so far has only been described in rats [41] and in the human brain [37]. The TagRFP and thus P2RY1 gene expression within the olfactory bulb are in agreement with the known expression pattern of the P2Y_1_R in the olfactory bulb as assessed by functional analysis [43]. We found P2RY1 gene expression in dopaminergic neurons of the VTA, which fits well to functional data showing that stimulation of the P2Y_1_R expressed in the VTA resulted in the increased release of glutamate [44] and neuronal fiber growth [45].

#### Other transgenic models

A BAC transgenic tool with which to study P2X4R-expressing cells is P2RX4 reporter mice Tg(P2RX4-tdTomato)1Khakh expressing the red fluorescent protein tdTomato under the control of the P2X4 locus by insertion into exon 1 of the P2RX4 gene to replace the endogenous initiation of translation codon in the BAC clone RP23-448O6 [46]. The pattern of tdTomato expression in the brain of these mice matched P2X4 mRNA distribution as described in the Allen Brain Atlas database. Furthermore, it highlights the role of well-characterized reporter mice to reveal new insights into purinergic receptor roles as the discovery of feeding-related regulation of presynaptic P2X4R responses in the hypothalamic arcuate nucleus.

The P2RX7 reporter mice Tg(P2RX7-EGFP)FY174Gsat/Mmcd from the aforementioned GENSAT project express an enhanced green fluorescent protein (EGFP) reporter gene driven by the regulatory sequences of the P2RX7 gene on the BAC clone RP23-181F3. The reporter gene was inserted at the initiating ATG codon of the first coding exon of the P2RX7 gene and is followed by a polyadenylation sequence. These mice were used as a tool to show that expression of the receptor is selectively increased in CA1 pyramidal and dentate granule neurons, as well as in microglia in mice that developed a status epilepticus after intra-amygdala injection of kainic acid [47]. However, these GENSAT P2X7 GFP-reporter mice were recently shown to exhibit prominent ectopic expression [48], which makes the evaluation of the P2X7 expression by these mice at least difficult or even error-prone.

In another study, BAC transgenic mice were generated for the in vivo analysis of the P2X7R expression [48]. Within the CNS, the P2X7R plays a role in several brain diseases such as epilepsy and Alzheimer’s disease, and since its identification, researchers still debate whether P2X7Rs are expressed in neurons and in other types of brain cells, although an increasing number of studies suggest its presence in neurons [49]. To resolve this issue and to provide a model for in vivo analysis of P2X7Rs P2X7 BAC transgenic mice were generated that overexpress GFP-tagged P2X7 under the control of a BAC-derived mouse P2X7 gene (P2rx7) promoter [48]. Under physiological conditions in the CNS, the P2X7R was shown to be predominantly located in microglia and oligodendrocytes a. After neural tissue damage or following status epilepticus, no upregulation of P2X7 protein in neurons was observed. Thus, the authors concluded that the reported P2X7-dependent neuronal damage may be the consequence of the pronounced manifestation of microglia activation rather than the direct activation of neuronal P2X7Rs [48].

Another reporter mouse line developed as a knock-in mouse that is expressing Cre recombinase in a P2X2-dependent fashion and subsequently crossed with a cre-sensitive tdTomato mouse line [39]. Moreover, similar to our findings, this mouse line shows reporter expression in neurons in the dentate gyrus within the hippocampus. Another mouse line indicates the expression of P2X2Rs in hippocampal neurons, using the genetically encoded fluorescent indicator for Ca^2+^ YC3.1 as a fusion P2X2 receptor (P2X2-YC) recombined into the BAC vector RP23-221H5 [40]. With this Tg(Camk2a-P2rx2/YC3.1)21Khakh, mouse line-specific P2X2-YC expression was observed within mossy fibers, particularly in the stratum lucidum of the hippocampus, generating a tool to drive neurotransmitter release selectively from the mossy fiber pathway. This fluorescently tagged receptor expressed in hippocampal neurons has already been used successfully to track its activation in vivo [50], and these mice may be useful to get a deeper insight into mossy fiber physiology.

Additionally, a P2RY1 knockout with lacZ reporter (P2RY1^tm1(KOMP)Vlcg^) produced by Regeneron Pharmaceuticals (Tarrytown, NY, USA) for the knockout mouse project (KOMP) at the University of California is meanwhile available and was recently used to study P2Y_1_R expression in the developing cochlea [51]. It was shown that burst firing of mouse inner hair cells prior to hearing onset requires P2RY1 autoreceptors expressed by inner supporting cells and that purinergic signaling in cochlear supporting cells regulates hair cell excitability by volume control of the extracellular space [51].

Taken together, our P2X2R and P2Y_1_R mouse models and other P2R transgenic models designed for visualizing the P2R expression have advanced the understanding of purinergic transmission, but BAC transgenic models appeared not always to be straightforward.

### Limitations and pitfalls in expression analysis with BAC transgenic mice 

BAC transgenic mice offer some advantages over conventional knock-in approaches. The enhancement of expression as a result of the random integration of multiple copies of the transgene, avoiding integration site artifacts and using the endogenous regulatory elements of the genes of interest and not least the comparatively fast generation of these transgenic mice (at least at that time), is certainly of great advantage. This has already been described frequently [22]. On the other hand, although limitations have been described (for instance see [22]), reports on various other problems are sparse. One of the most frequently described issues in BAC trangenesis is the unwanted effects caused by integration of the large transgene in another gene. This can, for example, lead to a functional knockout of the affected gene or cause a changed expression pattern of the gene [22]. This may lead to misinterpretation regarding the effect of the transgene expression. In order to rule out that phenotypes are due to positional effects or disruption of endogenous genes at the integration site, it is generally recommended to analyze at least two transgenic mouse lines for a given transgene with their random and unique genomic integration sites of the BAC transgene. Only reproducible phenotypes between the different lines should be unambiguously attributed to the transgene expression [22].

In the present paper, we have taken the opportunity to share also openly our own experiences and problems for the benefit of other scientists. Even though the figures above are showing promising results and have brought in part the hoped-for gain in knowledge regarding the expression analysis of P2X2 or P2Y_1_ receptors, the projects have subsequently encountered considerable challenges.

In the P2X2R project, the native TagRFP fluorescence was weak and hardly detectable, and even after using using anti-TagRFP immunostaining, additional signal amplification systems were necessary to detect mP2X2RTagRFP expression. Thus, except for the primarily cultured DRG neurons, it was not possible to reliably detect the expression of the mP2X2-TagRFP transgene in living cells from analyzed tissue. The preceding comprehensive and diligent analyses of P2X2 constructs in heterologous expression systems (*Xenopus oocytes* and HEK293 cells), confirming that TagRFP fusion did not markedly affect assembly, expression and function, were not able to predict strong native fluorescence in P2X2-expressing cells in the transgenic animal when expression is driven by the endogenous regulatory elements of the P2RX2 gene. Of course, this is not generally unexpected, but once again, this shows that results from overexpression experiments may not reflect the situation in vivo. For this reason alone, projects to generate (BAC) transgenic mice always remain risky.

One problem that occurred in the P2Y_1_R project in the further course of breeding is the intermittently occurring low fluorescence observed in some transgenics during breeding and the successively weaker fluorescence in the course of further generations until after about 2 years of breeding no fluorescence was detectable in neuronal processes, and also the cell bodies showed only a weak residual labeling (c.f. Figure 6). Also, over time, the fluorescence became increasingly granular in the cell bodies. As a likely explanation, the initial strong fluorescence was caused by the integration of several copies of the transgene possibly at more than one site into the genome. During the course of breeding, individual copies of the transgene were lost, resulting in a decrease of the expression of the TagRFP reporter. In the SOD1 mouse model of amyotrophic lateral sclerosis, it was already shown that a sporadic loss of transgene copy number can occasionally occur, probably by internal recombinations. The sporadic deletion of integrated transgene copies in the course of the breeding clearly reduced transgene expression as compared to expression in preceding generations prior to deletion [52]. Conventional PCR used for genotyping is not able to detect the number of copies, so that a loss of individual copies or the loss at one of multiple integration sites during the breeding process remains undetected. Thus, intermittent southern blot analysis or quantitative PCR techniques are suitable to quantify the number of copies and can help to detect this problem early and to adjust the breeding process if necessary. Monitoring of the BAC transgene copy number should be performed on a regular basis in the course of breeding to evaluate transgene expression and to confirm stability of integrations and germline transmission across successive generations.

In the future, however, BAC recombineering may no longer be the first choice for genome editing, but transgenes will directly enter endogenous loci through more versatile and adaptable nuclease-mediated genome-editing methods such as the CRISPR/Cas technology, which have developed rapidly over the last decade.

## Conclusion 

Our P2X2R and P2Y_1_R mouse models and other P2R transgenic models designed for visualizing the P2R expression have advanced the knowledge toward a deeper understanding of P2R expression and purinergic transmission, but BAC transgenic models appeared not always to be straightforward and permanent reliable.

## Supplementary Information

Below is the link to the electronic supplementary material.

Suppl. Fig. 1 Screening regarding TagRFP expression in hippocampal cryosections of offspring of different transgenic P2X2R mice lines (founder lines) C57BL/6J-Tg(RP23-333M22P2X2-StrepHis-TagRFP). Confocal fluorescence imaging of PFA-fixed coronal hippocampus cryosections of the eight fertile P2X2-TagRFP-BAC founder lines (**A–H**) and a wild-type mouse (WT) (**I**). Shown is an overlay of TagRFP-immunofluorescence(red) and DAPI stained nuclei (blue) in the cornu ammonis (CA) and dentate gyrus of the hippocampus. All mice were 6months old. (A–I) Scale bar: 50µm; *dg* dentate gyrus, *sgz* subgranularzone, *ml* molecular layer (PDF 9543 KB)

Suppl. Fig. 2 Screening regarding native TagRFP fluorescence in hippocampal cryosections of offspring of different transgenic P2Y_1_R mice lines (founder lines) C57BL/6J-Tg(RP23-452G4P2RY1-TagRFP). Confocal fluorescence imaging of PFA-fixed coronal hippocampus cryosections of the eight fertile TagRFP-P2Y1-BAC founder lines (**A–H**) and a wild-type mouse (WT) (**I**). Shown is an overlay of the native TagRFP-fluorescence (red) and DAPI stained nuclei (blue) in the cornu ammonis (CA) 1 and 2 of the hippocampus. All mice were 6months old. (A–I) Scale bar: 50µm; *so* stratum oriens, *sp* stratum pyramidale, *sr* stratum radiatum (PDF 5106 KB)

## Data Availability

The data that support the findings of this study are available from the corresponding author upon reasonable request.
