# Supplementary material for: BAC transgenic mice to study the expression of P2X2 and P2Y1 receptors
Source: Purinergic Signal. 2021 May 28;17(3):449–65. doi: 10.1007/s11302-021-09792-9 (PMC8410928; doi:10.1007/s11302-021-09792-9)
Supplement: Supplementary file 1 — Screening regarding TagRFP expression in hippocampal cryosections of offspring of different transgenic P2X2R mice lines (founder lines) C57BL/6J-Tg(RP23-333M22P2X2-StrepHis-TagRFP). Confocal fluorescence imaging of PFA-fixed coronal hippocampus cryosections of the eight fertile P2X2-TagRFP-BAC founder lines (A–H) and a wild-type mouse (WT) (I). Shown is an overlay of TagRFP-immunofluorescence(red) and DAPI stained nuclei (blue) in the cornu ammonis (CA) and dentate gyrus of the hippocampus. All mice were 6months old. (A–I) Scale bar: 50µm; dg dentate gyrus, sgz subgranularzone, ml molecular layer (PDF 9543 KB) [file 11302_2021_9792_MOESM1_ESM.pdf]

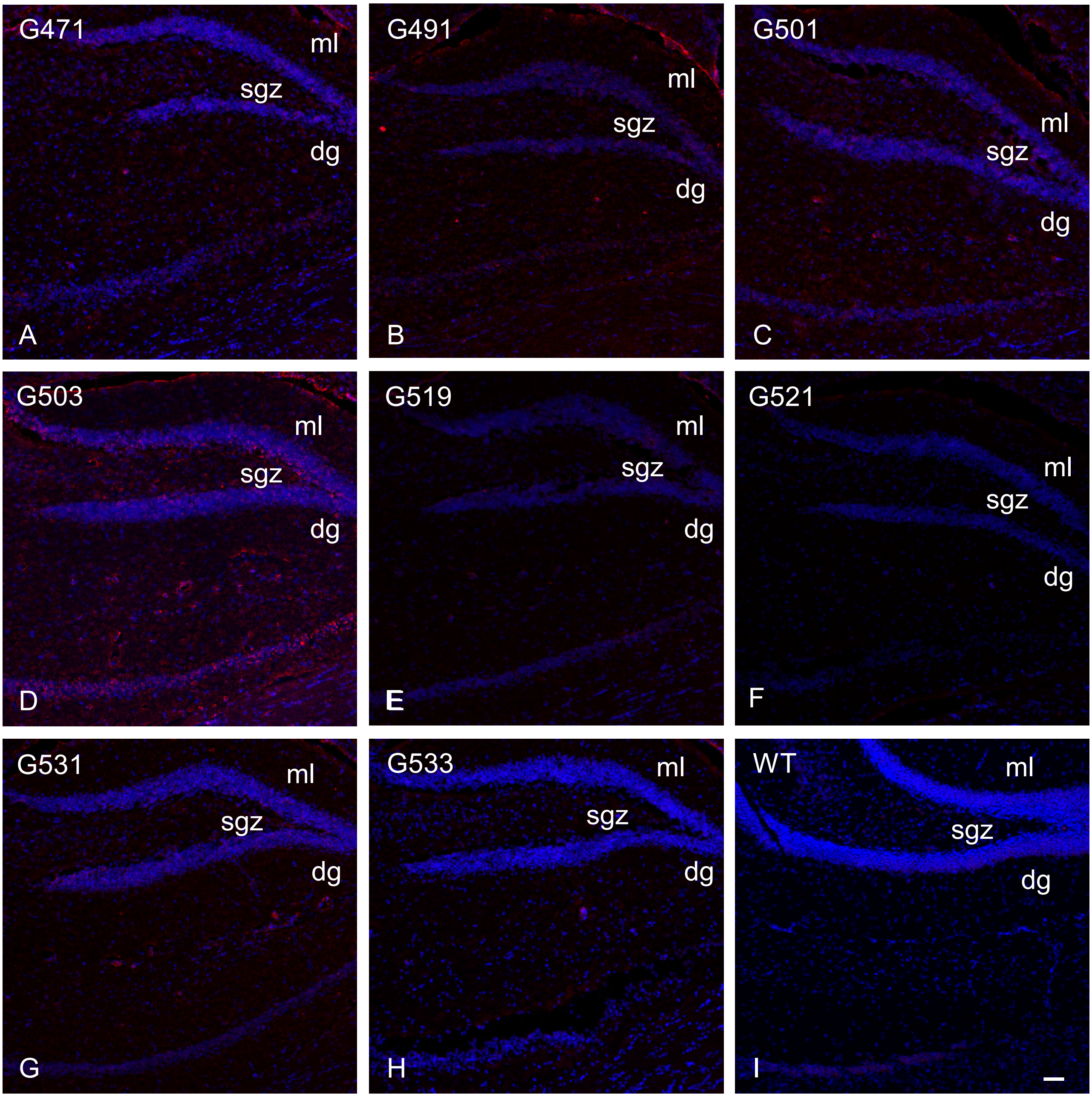

Suppl. Fig. 1. Screening regarding TagRFP expression in hippocampal cryo-sections of offspring of different transgenic P2X2R mice lines (founder lines) C57BL/6J-Tg (RP23-333M22P2X2-StrepHis-TagRFP)
Confocal fluorescence imaging of PFA-fixed coronal hippocampus cryosections of the eight fertile P2X2-TagRFP-BAC founder lines (A-H) and a wild-type mouse (WT) (I). Shown is an overlay of TagRFP-immunofluorescence (red) and DAPI stained nuclei (blue) in the cornu ammonis (CA) and dentate gyrus of the hippocampus. All mice were 6 months old. (A-I) scale bar: 50 µm; dg: dentate gyrus, sgz: subgranular zone, ml: molecular layer.
