# Supplementary material for: BAC transgenic mice to study the expression of P2X2 and P2Y1 receptors
Source: Purinergic Signal. 2021 May 28;17(3):449–65. doi: 10.1007/s11302-021-09792-9 (PMC8410928; doi:10.1007/s11302-021-09792-9)
Supplement: Supplementary file 2 — Screening regarding native TagRFP fluorescence in hippocampal cryosections of offspring of different transgenic P2Y1R mice lines (founder lines) C57BL/6J-Tg(RP23-452G4P2RY1-TagRFP). Confocal fluorescence imaging of PFA-fixed coronal hippocampus cryosections of the eight fertile TagRFP-P2Y1-BAC founder lines (A–H) and a wild-type mouse (WT) (I). Shown is an overlay of the native TagRFP-fluorescence (red) and DAPI stained nuclei (blue) in the cornu ammonis (CA) 1 and 2 of the hippocampus. All mice were 6months old. (A–I) Scale bar: 50µm; so stratum oriens, sp stratum pyramidale, sr stratum radiatum (PDF 5106 KB) [file 11302_2021_9792_MOESM2_ESM.pdf]

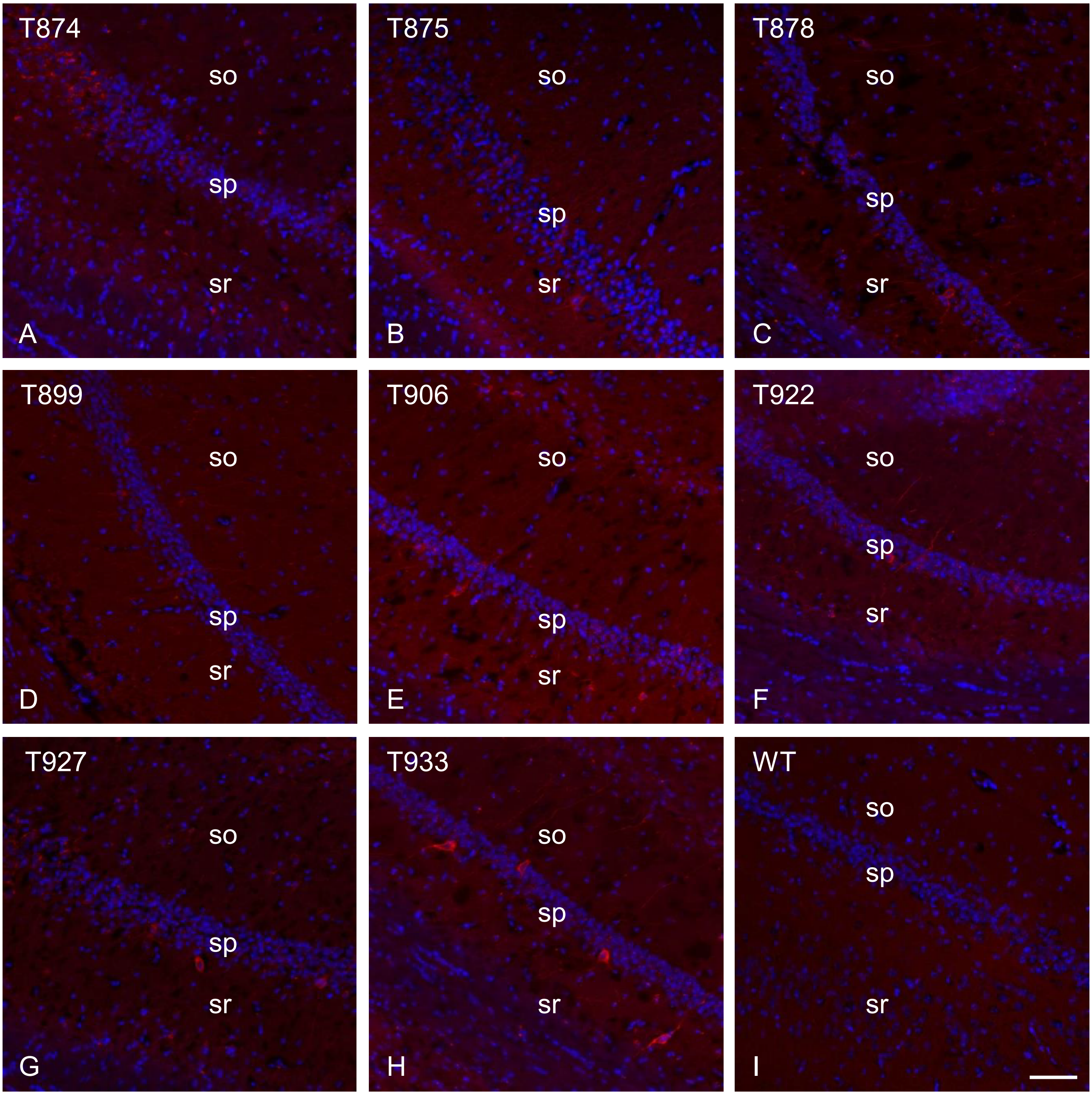

Suppl. Fig. 2. Screening regarding native TagRFP fluorescence in hippocampal cryo-sections of offspring of different transgenic P2Y1R mice lines (founder lines) C57BL/6J-Tg(RP23-452G4P2RY1-TagRFP)
Confocal fluorescence imaging of PFA-fixed coronal hippocampus cryosections of the eight fertile TagRFP-P2Y1-BAC founder lines (A-H) and a wild-type mouse (WT) (I). Shown is an overlay of the native TagRFP-fluorescence (red) and DAPI stained nuclei (blue) in the cornu ammonis (CA) 1 and 2 of the hippocampus. All mice were 6 months old.
(A-I) scale bar: 50 µm; so: stratum oriens, sp: stratum pyramidale, sr: stratum radiatum.
